# Application Value of Color Doppler Ultrasound and Ultrasound Contrast in the Differential Diagnosis of Ovarian tumor

**DOI:** 10.12669/pjms.36.2.847

**Published:** 2020

**Authors:** Haijing Zhang, Jinming Wang, Rui Guo

**Affiliations:** 1 Haijing Zhang, Department of Ultrasonic Medicine, Binzhou People’s Hospital, Shandong, 256610, China; 2 Jinming Wang, Department of Ultrasonic Medicine, Binzhou People’s Hospital, Shandong, 256610, China; 3 Rui Guo, Department of Ultrasonic Medicine, Binzhou People’s Hospital, Shandong, 256610, China

**Keywords:** Color Doppler ultrasound, Differentiation, Ovarian tumor, Ultrasound contrast

## Abstract

**Objective:**

To study the value of color Doppler ultrasound and ultrasound contrast in differential diagnosis of ovarian tumors.

**Methods:**

Ninety-six patients with ovarian tumors who were treated in our hospital from May 2017 to July 2018 and confirmed by pathological examination were selected as the research subjects. All patients were examined by color Doppler ultrasound and ultrasound contrast. The sensitivity, specificity and accuracy of the two methods were compared, and the parameters of ultrasound contrast in the diagnosis of benign and malignant tumors were observed and compared.

**Results:**

The sensitivity, specificity and accuracy of ultrasound contrast in the diagnosis of ovarian tumors were higher than those of color Doppler ultrasound (P<0.05). There were significant differences in the time of initiation enhancement, time to peak and perfusion intensity in the diagnosis of benign and malignant lesions by ultrasound contrast (P<0.05).

**Conclusion:**

In the differential diagnosis of ovarian tumors, ultrasound contrast has more advantages than color Doppler ultrasound in displaying the blood perfusion information of tumors. It has high diagnostic accuracy and clinical application value.

## INTRODUCTION

Ovarian tumor is one of the reproductive system diseases with a high incidence in women, which can be divided into benign and malignant tumors. The incidence of malignant ovarian tumor ranks the second place among female reproductive system tumors, and its mortality rate ranks the first. The latest statistics show that the incidence of ovarian tumors is increasing in recent years, which seriously threatens the health and life safety of women.^1^-^3^ For early ovarian cancer, the curative effect of surgical radical and chemical treatment is good, but the 5-year survival rate of patients with ovarian cancer is still not significantly improved; the main reason is that more than 70% of ovarian cancer cases has been at the late stage at the time of diagnosis. For patients with advanced ovarian cancer or distant metastasis, the curative effect of surgical radical and chemical treatment is poor.^4^ Therefore, early diagnosis of ovarian cancer is a key issue to improve survival rate. The clinical symptoms of ovarian cancer patients are not obvious in the early stage. Lumbar acid, abdominal distension, abdominal pain, frequency of urination, vaginal bleeding and abdominal mass are the main clinical manifestations. The causes of ovarian cancer are complex, so it is still difficult to diagnose and differentiate early ovarian cancer histologically.^5^ In the past, ovarian tumors were found mainly by conventional gynecological examination or general examination, with a high rate of missed diagnosis. Many patients have had distant metastasis when they visited the doctor, missing the best treatment time.^6^ At present, many researchers are devoted to exploring ovarian tumor markers and imaging technologies with high sensitivity and specificity and creating a “golden standard” for early diagnosis, prognosis analysis and monitoring of ovarian cancer to determine the best treatment method for patients.^7^,^8^ In this study, 96 patients with ovarian tumors who were admitted to our hospital were selected as the research subject. The purpose of this study was to compare the clinical value of color Doppler ultrasound and ultrasound contrast in the differential diagnosis of ovarian tumors.

## METHODS

Ninety-six patients with ovarian tumors who were treated in our hospital from May 2017 to July 2018 were selected as the study subjects. The age of the patients ranged from 21 to 72 years, with an average of (51.65±3.87) year. Among them, 22 cases were found to have ovarian tumors, 51 cases were found to have abdominal pain, 16 cases were found to have irregular menstruation with vaginal bleeding, and seven cases were found to have changed defecation habits and unintentional palpation of tumors. Inclusive criteria included 75 over years old, being diagnosed histopathologically as primary benign or malignant ovarian tumors, receiving no other treatment before this visit, and having complete clinical, imaging and laboratory data. Exclusion criteria were having pelvic congenital malformation; having ovarian metastatic tumors; having acute inflammation; being in pregnancy and lactation period; having liver and kidney insufficiency, heart failure and hematological diseases; unable to cooperate with researchers. This study was approved by the ethics committee of the hospital, and all patients signed informed consent.

Color Doppler ultrasound examination method: The Logiq7 color Doppler ultrasound diagnostic instrument (GE, USA) was used; a broadband linear array probe was used, and the center frequency was set as 7.5 MHz. The patients took a supine position, and the ovarian parts of the patients were scanned. The size, boundary, capsule and echo of ovarian lesions were observed and recorded. The distribution of blood vessels and blood flow in the lesions were analyzed using Doppler flow imaging.

Sono Vue was used as contrast agent. The prepared sulfur hexafluoride suspension was intravenously injected into the patient from the center of elbow, and then sodium chloride injection was used for washing. The contrast lasted for 5 minutes, and the images were stored and analyzed by TIC software. The enhancement and regression of the contrast agent in tumors was observed using ultrasound equipment, and the microcirculation perfusion status and parameters of tumors were observed.

Diagnostic criteria for benign and malignant tumors by color Doppler ultrasound were as follows.^9^ Grading was mainly based on the characteristics of blood flow morphology. A tumor was evaluated as grade 0 if no blood flow signals were found in the tumors, as Grade-I if there were transient blood flow signals or occasional blood vessels in the tumors, as Grade-II if there were multiple blood flow signals in the tumors or clear blood vessels passing through the lesions, and as Grade-III if there were a large number of blood flow signals in the tumors or large blood vessels passing through the lesions. Tumors with grade 0~I and regular blood vessels were evaluated as benign tumors, and tumors with Grade-II~III and complex and dilated blood vessels were evaluated as malignant tumors.

Diagnostic criteria of ultrasound contrast for benign and malignant tumors were as follows.^10^ According to the morphological characteristics of blood flow, tumors could be divided into four grades. A tumor was evaluated as grade 0 if there was no enhancement inside the mass and there was homogeneous enhancement outside the mass, as Grade-I if the wall, segmental space and solid area of the mass had enhancement, there was no obvious curvature of the blood vessels, and the density the enhancement area was even, as Grade-II if the wall, segmental space and solid area of the mass had enhancement, distorted vascular bundles appeared, and the density of the enhancement area was uneven, as Grade-III if there were a large number of disordered and distorted blood vessels in the solid area of the mass and the density of the enhancement area was extremely uneven. Tumors with grade 0 ~ I was evaluated as benign tumors, and tumors with Grade-II and III were evaluated as malignant tumors.

The diagnostic results of color Doppler ultrasound and ultrasound contrast were observed, and the sensitivity, specificity and accuracy were calculated: sensitivity=number of true positive cases/(number of true positive cases + number of false negative cases)×100%, specificity=number of true negative cases/(number of false positive cases+ number of true negative cases)×100% and accuracy=number of true positive cases +number of true negative cases/total number of cases×100%. The parameters of ultrasound contrast in diagnosing benign lesions and malignant lesions were compared, including time of initiation enhancement, time to peak and perfusion intensity.

### Statistical analysis

SPSS 20.0 was used for data analysis. The measurement data were expressed by mean ± standard deviation and processed by t-test. The categorical data were processed by Chi-square test. The difference was thought as statistically significant if P<0.05.

## RESULTS

### Postoperative pathological diagnosis

Sixty-six cases of benign tumors were found after pathological diagnosis, including 10 cases of endometriotic cyst, 8 cases of mature teratoma, 10 cases of serous cystadenoma, 32 cases of simple cyst and 6 cases of salpingitis; 30 cases of malignant tumors were found by pathological diagnosis, including eight cases of granulosa cell tumors, 5 cases of metastatic tumor, seven cases of serous cystadenoma, seven cases of mucinous cystadenocarcinoma and 3 cases of theca cell tumors.

### Comparison of diagnostic results between two methods

Color Doppler ultrasound confirmed 52 cases of benign tumors, and there were 12 cases of misdiagnosis and two cases of missed diagnosis; it confirmed 23 cases of malignant tumors, and there were six cases of misdiagnosis and one case of missed diagnosis. Ultrasound contrast confirmed 64 cases of benign tumors, and there were one case of misdiagnosis and one case of missed diagnosis; it confirmed 28 cases of malignant tumors, and there were one case of misdiagnosis and one case of missed diagnosis. The sensitivity, specificity and accuracy of ultrasound contrast were higher than those of color Doppler ultrasound, and the difference was statistically significant (P<0.05, Table-I).

**Table-I:** Diagnostic results between two methods [n (%)].

Examination method	Accuracy	Sensitivity	Specificity
Ultrasound contrast	92(95.83)	64(96.97)	28(93.33)
Color Doppler ultrasound	75(78.13)	52(78.79)	23(76.67)
X^2^	12.183	9.154	8.657
P	<0.05	<0.05	<0.05

The time of initial enhancement and time to peak of benign ovarian tumors were higher than those of malignant ones (P<0.05), and the perfusion intensity of benign lesions was lower than that of malignant ones (P<0.05, Table-II).

**Table-II:** Ultrasound contrast parameters in diagnosing benign and malignant ovarian lesions.

Examination method	Time of initial enhancement (s)	Time to peak	Perfusion intensity (dB)
Benign lesions (n=66)	25.73±2.31	27.93±5.17	6.06±3.28
Malignant lesions (n=30)	11.05±1.36	18.16±4.26	18.83±2.34
X^2^	14.763	7.539	11.728
P	<0.05	<0.05	<0.05

## DISCUSSION

Early detection and correct benign and malignant determination of ovarian tumors have very important clinical significance.^11^ However, due to the complexity of ovarian histological types and the particularity of anatomical location, the early diagnosis and differential diagnosis of ovarian tumors are still insufficient and lack of mature early diagnosis methods, so it is still difficult to diagnose and differentiate early ovarian tumors histologically.^12^ At present, ultrasound has become a conventional way to detect ovarian tumors because of its convenience, non-invasive, intuitive and good repeatability. The commonly used new ultrasound technologies include Doppler ultrasound, three-dimensional ultrasound, and ultrasound contrast and so on.

The diagnosis of benign and malignant ovarian lesions is based on the theory that the formation of neovascularization is the premise and basis of tumorigenesis.^13^ Color Doppler ultrasound has high image resolution and wide scanning range, and its unique probe can improve the differential diagnosis rate of benign and malignant lesions in abdomen and superficial organs.^14^,^15^ The application of color Doppler ultrasound in the differential diagnosis of ovarian tumors can clearly show the pelvic organs and lesion sites to obtain good imaging of the hemodynamics and vascular distribution around the tumors. However, it has obvious limitations on the display of small blood vessels, low-velocity blood flow or blood flow in deep tumors.^16^,^17^

Ultrasound contrast is based on the conventional color Doppler ultrasound, which has more advantages in revealing the blood perfusion information of tumors.^18^ It is different from color Doppler examination as it is not affected by breathing, movement and angle and can display small vessels with diameter less than 200 μm. Therefore, it can display the blood supply around and inside lesions, especially the small vessels with low velocity and also show the intensity of perfusion in different areas of the lesion and the sequence of perfusion and regression.^19^ Contrast agent can effectively enhance two-dimensional ultrasound image and Doppler flow signal through its strong scattering effect in blood flow to fully display the blood flow signal in lesions, including small blood vessels in tumors, low velocity blood flow or blood flow distribution in the deep part of the tumor and accurately evaluate the blood vessels in the tumor. It overcomes the influence of the size and depth of tumors on the blood flow display rate, achieves the purpose of differential diagnosis, abd improves the accuracy of tumor diagnosis.^20^

The parameters of ultrasound contrast are closely related to the blood perfusion of tumor tissues. The time of initial enhancement reflects the time when contrast agents appear in the tumor body. The peak intensity and time to peak reflect the maximum amount of contrast microbubbles in the vascular bed and the perfusion time needed to reach the maximum amount respectively.^21^ A previous study has shown that the ultrasound contrast of malignant tumors generally presents a pattern of fast forward and slow regression, while benign tumors mostly present a pattern of slow forward and slow regression.^22^ The results of this study showed that the time of initial enhancement, time to peak and perfusion intensity of malignant ovarian tumors were significantly larger than those of benign tumors. It was because the blood vessels of malignant ovarian tumors increased and distorted to form vascular loops or networks, and the microbubbles in the local blood vessels of the lesions accelerated after the injection of the contrast agent, resulting in fast penetration of the contrast agent and abrupt ascending branch.

Therefore, ultrasound contrast technology can effectively identify the benign and malignant nature of ovarian tumors, especially for those patients with new capillaries or slow blood flow in malignant tumors which cannot be detected by color Doppler. It can provide help for the early diagnosis of ovarian tumors and make up for the limitations of two-dimensional and color flow imaging in the detection of low-velocity blood flow and small blood vessels in ovarian tumors. The results of this study showed that ultrasound contrast was superior to color Doppler ultrasound in sensitivity, specificity and accuracy in the diagnosis of ovarian tumors, suggesting that ultrasound contrast was more effective than color Doppler ultrasound in the differential diagnosis of ovarian tumors, which is similar to the results of Kumazawa.^23^

## CONCLUSION

In conclusion, compared with color Doppler ultrasound, ultrasound contrast holds higher diagnostic accuracy in differential diagnosis and precise characterization of ovarian tumors. It can help diagnose early and facilitate in outlining the roadmap for treatment, therefore, increases the survival time of patients.

### Authors’ Contribution:

**HJZ & JMW:** Study design, data collection and analysis.

**JMW & RG:** Manuscript preparation, drafting and revising.

**HJZ:** Review and final approval of manuscript.
